# Estimating the prevalence of hepatitis C among intravenous drug users in upper middle income countries: A systematic review and meta-analysis

**DOI:** 10.1371/journal.pone.0212558

**Published:** 2019-02-26

**Authors:** Víctor Granados-García, Yvonne N. Flores, Lizbeth I. Díaz-Trejo, Lucia Méndez-Sánchez, Stephanie Liu, Guillermo Salinas-Escudero, Filiberto Toledano-Toledano, Jorge Salmerón

**Affiliations:** 1 Unidad de Investigación Epidemiológica y en Servicios de Salud Área Envejecimiento, Centro Médico Nacional Siglo XXI, Instituto Mexicano del Seguro Social (IMSS), Ciudad de México, México; 2 Unidad de Investigación Epidemiológica y en Servicios de Salud, Delegación Morelos, Instituto Mexicano del Seguro Social, Cuernavaca, Morelos, México; 3 UCLA Department of Health Policy and Management, Fielding School of Public Health and Jonsson Comprehensive Cancer Center, Los Ángeles, CA, United States of America; 4 Centro Nacional de Programas Preventivos y Control de Enfermedades, Secretaría de Salud, Ciudad de México, México; 5 Unidad de Epidemiología Clínica, Hospital Infantil de México Federico Gómez Instituto Nacional de Salud, Ciudad de México, México; 6 Facultad de Medicina, Universidad Nacional Autónoma de México, Ciudad de México, México; 7 University of Washington, Department of Epidemiology, School of Public Health, Seattle, WA, United States of America; 8 Centro de Estudios Económicos y Sociales en Salud, Hospital Infantil de México Federico Gómez, Ciudad de México, México; 9 Unidad de Investigación en Medicina Basada en Evidencias, Hospital Infantil de México Federico Gómez Instituto Nacional de Salud, Ciudad de México, México; 10 Centro de Investigación en Políticas, Población y Salud, Facultad de Medicina, Universidad Nacional Autónoma de México, Ciudad de México, México; 11 Centro de Investigación en Salud Poblacional, Instituto Nacional de Salud Pública, Cuernavaca, México; University of Toronto, CANADA

## Abstract

**Aim:**

This systematic review and meta-analysis characterizes the prevalence of hepatitis C virus (HCV) infection among intravenous drug users (IDUs) in upper middle-income countries.

**Methods:**

Five databases were searched from 1990–2016 for studies that took place in countries with a GDP per capita of $7,000 to $13,000 USD. The data extraction was performed based on information regarding prevalence, sample size, age of participants, duration of intravenous drug use (IDU), recruitment location, dates of data collection, study design, sampling scheme, type of tests used in identifying antibody reactivity to HCV, and the use of confirmatory tests. The synthesis was performed with a random effects model. The Cochrane statistical Q-test was used to evaluate the statistical heterogeneity of the results.

**Results:**

The 33 studies included in the analysis correspond to a sample of seven countries and 23,342 observations. The point prevalence value estimates and confidence intervals of the random effects model were 0.729 and 0.644–0.800, respectively for all seven countries, and were greatest for China (0.633; 0.522–0.732) as compared to Brazil (0.396; 0.249–0.564). Prevalence for Montenegro (0.416; 0.237–0.621) and Malaysia (0.475; 0.177–0.792) appear to be intermediate. Mexico (0.960) and Mauritania (0.973) had only one study with the largest prevalence. A clear association was not observed between age or duration of IDU and prevalence of HCV, but the data from some groups may indicate a possible relationship. The measures of heterogeneity (Q and I2) suggest a high level of heterogeneity in studies conducted at the country level and by groups of countries.

**Conclusions:**

In this systematic review and meta-analysis, we found that the pooled prevalence of HCV was high (0.729) among a group of seven upper middle income countries. However, there was significant variation in the prevalence of HCV observed in China (0.633) and Brazil (0.396).

## Introduction

Infection with the hepatitis C virus (HCV) is a serious public health problem due to its association with diseases like chronic hepatitis, cirrhosis, and hepatocellular carcinoma [1–4]. Evidence suggests that the prevalence and number of infected patients has decreased in higher income countries [5]. On the other hand, this disease has increased significantly in certain low-income countries in Africa and Asia [6]. Intravenous drug users (IDU) are one of the groups with a higher prevalence of HCV infection. This group has a significantly higher risk of infection compared with non-injection drug users, or individuals who do not use illegal drugs, due to the sharing of contaminated needles [7–11]. This has been documented in a number of systematic reviews that report the prevalence of HCV among IDUs, which have mostly focused on European countries [7, 12–19]. Other reviews have examined specific countries or regions like China, Latin America, Iran, Australia, the Middle East, and North Africa [20–24]. Of the eight reviews that have been published, four were conducted worldwide and the other four represent middle and low-income countries. In some instances, the country-specific prevalence data reported in the worldwide reviews is limited and it is not clear how the authors determined the point or interval estimates [14–16, 19]. Additionally, it can be difficult to assess how certain estimates of HCV prevalence were determined since the sources reported in web-appendices are not always available, especially for the older publications [14].

Also relevant, is the fact that more studies on IDUs have been conducted in high-income countries, which may suggest that high-income countries have a larger proportion of IDUs. An alternative explanation could be that some high-income countries are also likely to have a larger budget to estimate the prevalence of HCV infection among IDUs. Conversely, research regarding the prevalence of HCV infection in middle-middle and low-middle income countries is limited to a few countries [20].

Estimating the national prevalence of HCV infection among high-risk individuals, such as IDUs, in middle- and low-income countries is important to help guide interventions to reduce the burden of disease and its economic consequences. To the best of our knowledge, there are no systematic reviews that have investigated the prevalence of HCV infection among IDUs in upper-middle income countries. Therefore, our research question is what is the prevalence of HCV infection (measured by HCV antibodies) among IDU population in upper-middle-income countries? The purpose of this systematic review is to estimate the prevalence of HCV infection among IDUs in several upper-middle income countries (UMIC) and as a group, by means of meta-analysis techniques; and to provide an analysis of prevalence by age and duration of IDU use.

## Methods

An electronic literature search was conducted to identify English and Spanish articles published between 1990 and 2016 that reported the prevalence of HCV among IDUs in upper middle income countries. The following five databases were used to conduct this search: PubMed, SCOPUS, Medic Latina, LILACS and Scielo Citation Index (Thompson Reuters), and duplicate articles were eliminated. We used five Medical Subject Headings (Mesh): “Prevalence”, “Hepatitis C”, “Hepatitis C Antibodies”, “Substance-Related Disorders”, and “Substance Abuse and Intravenous”. We also used the following keywords: “prevalence”, “hepatitis C”, “HCV”, “drug abuse”, “drug users”, “prevalencia”, “abuso de drogas” and “VHC” (Table 1A). We did not review the articles listed in the reference sections of the manuscripts we identified. Our search strategy included 26 countries classified as upper middle income, with a gross domestic product (GDP) per capita income between $7,000 and $13,000 US dollars (USD) in 2015, as defined by the World Bank using the Atlas method [25].

**Table 1:** Characteristics of studies included in the meta-analysis that report HCV prevalence data among IDUs in upper middle income countries.

Study information		Study methodology			Results	
Study	Dates and site of recruitment and data collection (blood samples and tests and questionnaires)	Eligibility criteria (EC), sources and methods selection of participants (cross sectional)	Test Type (TT) and use of RNA reflex	Study size and analytical methods accounting for sampling strategy	Demographic (age, sex), clinical, social information of participants	Type drug, route of administration, time injecting drugs, risk behaviors
[30]	Dates not reported. Villages of farmers.	EC not reported. Participants were “Randomly selected”.	Anti-HCV Elecsys anti-HCV II assay. RNA reflex was not conducted.	Participants were randomly selected from 11 villages in Kuancheng county, Hebei province.	86%>40 years old, 70% males. 26 years from first injection in HCV positives.	Sodium benzoate or amphetamine. 26 years from first injection in HCV positives.
[31]	May-June 2012. Drop-in center of a needle-syringe exchange program. Different settings Detoxification, detention centers or MMT clinics and sentinel surveillance sites.	Participants were required to be Chinese and reside/work in Ruili > 6 months when interviewed. Biological and behavioral respondent-driven sampling.	ELISA / RNA reflex not conducted.	Convenience sampling was conducted. A biological and behavioral survey using respondent-driven sampling was conducted for more representativeness.	93% males, 50%>35 years old, 51% > 5 years in Anti-HCV positives.	Heroin, alcohol and amphetamine-type stimulants (ATS) were the most prevalent. Mean age at first use (no alcohol) 23.3 (SD = 3.7) years, with up to 26.4% starting before the age of 18 years.
[32]	2004–2012. National Methadone Database.	EC not reported. All data patients attending 11 methadone clinics were included in study.	Information on TT and RNA was not reported. Consider to exclude because not clear only IDUs are included in sample.	5,653 methadone patients were included in the prevalence estimate analysis.	Data on age, sex, and time injecting drugs was not provided.	No detailed information reported.
[33]	2006–2011. All entrants from 4 methadone maintenance treatment (MMT) centers fulfilling eligibility criteria.	EC: Entry to MMT clinic. Active IDU: Drug used (‘injecting’ or ‘mixed’), self-reported history of injecting or sharing syringes (30 days) and frequency >once in previous 30 days.	ELISA positive results were confirmed by Western blot and a 2nd ELISA.	Four MMT clinics were those with most service time, retained most complete records and are representative. Data collection was based on Comprehensive and self-administered questionnaire.	37%>40 years old, 89% males, 80% were IDU.	No detailed information reported on type and route of administration. ICD-10 diagnoses (opioid dependence). 67% >10 year in IDU. Data on sharing syringes and having sex without condom were reported.
[34]	Data collection was conducted in one medical center during 2012.	EC were not reported. From all 500 treated, 432 individuals in the district medical center participated in study.	Third generation Anti-HCV assay, RIBA was used to confirm positive Anti-HCV cases. RNA reflex was conducted	Details of study size and methods of sampling strategy were not reported.	Range 23–63 years, mean age 44 ±9 years old. 78% males.	Patients receive diaminon therapy for heroin addiction. TID: range 2–40 years, 15 ±5 years in overall IDU.
[35]	March 2009-october 2011. Communities, free HIV Voluntary Counseling and Testing (VCT), needle and syringe programs (NSP), or MMT.	EC were age ≥16 y, history of IDU, and able to provide informed consent. IDUS.	ELISA for anti-HCV / RNA reflex was not conducted	Sampling strategy details were not reported. Cross sectional study conducted with assistance of local Centers for Disease Control and Prevention.	Age participants 31.7 (31.4–32.0) years old. 93% males.	Type of drugs and route of administration not reported. Mean duration of drug injection 6.8 years. 88% > 5 years in HCV positives. Risk behaviors: duration of drug injection, needles/ syringes sharing, number of sexual partner and having a history of sexually transmitted diseases (STD).
[36]	Nov 2004- March 2008. Eight MMT clinics.	>18 y, met test DSM-IV (opiate dependence), willing to provide informed consent and blood collection. Exclusion if serious physical or mental illnesses or cognitive deficits. All drug users in Census with treatment in MMT clinics.	ELISA / RNA reflex was not conducted	All drug users who received treatment at clinics selected were investigated when admission to MMT program. Methods for sampling strategy were not reported.	Participants‘ age: 70%18–30, 63% 30–40, 49% 40–54 years old. 81% males.	Drug and route: Heroin injected. Non IDU also reported. Time of use: 1–2 years 0.07, 3–5 years 0.08, 5–9 years 0.35, 10–19 years 0.49 ≥20 years 0.01. Risk behaviors reported: Sharing injection, condom use, frequency with drug addict friends.
[37]	September 2009- December 2010. Compulsory and voluntary detoxification centers.	EC: >18 y, positive urine test for one (amphetamine type stimulant) ATS drug, and MDMA, at the time they entered center and signed informed consent.	ELISA was used to test HCV antibodies/ RNA reflex was not conducted.	Multicenter cross-sectional survey study. Details of sampling frame were not provided.	Participants‘ age: 37% 18–29 36% 30–39 27% 40–65. 66% males	Users ATS and IDU (ATS) (months) 15% 1–2 32% 3–12 51% 12–119 2% ≥ 120
[39]	March-September 2009. Community and MMT.	EC were not stated clear. Written informed consent and acceptance of questionnaire regarding history of intravenous drug usage and blood collection was required.	Anti-HCV test. RNA was not conducted.	Details on methods for sampling strategy were not provided. Chinese IDUS from Community and MMT program. Burmese IDUs from community.	Participants ‘age: China 32.3 (8.8 de), Burma 31.8 (9.8 de). China 99% males, Burma 96.5% males.	Specific details of drug and administration route were not reported. Years of DI: China 6.2 (4.8 de), Burma 4.2 (4.6 de).
[38]	2009–2010. One MMT Clinic from Shanghai and other from Kunming.	EC: opiate dependence (Criteria Diagnostic and Statistical Manual IV), ≥ 20 y and local residents. Not required to be sober, study activities (professionally trained clinical staff). All participants required WIC and were paid for their time.	No information on tests type was reported/ RNA reflex was not conducted.	Details on methods for sampling strategy were not provided. IDUS recruited from consecutive admissions (MMT programs).	Participants ‘age: 38.6 (7.9) 37.2 (8.3). Shanghai HCV+ 21% females, Kunming HCV+ 23.5%	75–85% of registered drug addicts use heroin and 50–70% of heroin users inject the drug. Years of DI: Shanghai HCV+ 12.2 (4.1 sd), Kunming HCV+ 12.8 (6.7 sd)
[40]	May-June 2008 and Oct-Dec 2008. Mandatory detoxification centers (MDCs)	EC: provide WIC, ≥18 y o, history of DU. Exclusion: serious physical or mental illness or intelligence deficits.	Antibody test not specific. RNA was not conducted.	Focus groups to assess feasibility and support of research among DU. physician assessed DU for eligibility and interests.	Median age 31 (range 24–38). 91.8% males. Prevalence of HIV, HBV, HCV, and syphilis.	62.5% IDU had injected ≥ 5 years. Risk behaviors reported: had sex with non-regular partners, low use of condom with non-regular partners. 21.65% male participants purchased sex and 34.4% females involved in providing commercial sex.
[44]	August-October 2006. Liuzhou Methadone Clinic.	Detailed EC were not reported. 597 IDU were recruited through street outreach, targeted advertising, and chain referral by the Guangxi Center for Disease Control and Prevention.	Anti-HCV was determined by ELISA/ RNA reflex was not conducted.	Study participants receive 80 Chinese Yuan ($10.00) for each follow-up visit to compensate for travel, time, and a meal. Authors consider unknown extent of generalization to other IDUs.	HCV positives %84 ≥41 years old. HCV positives %67 males.	Drug and route: Heroin, injected. 19% Use injection >11 years. Risk factors were needle use (cleaning), sharing (yes/no, frequency). Sexual active (yes/no).
[45]	Dates of field work for blood sampling not reported. Information on recruitment sites was not reported.	EC not reported. Details of random selection not reported.	HCV antibody presence by ELISA. RNA reflex was conducted and HCV mono-infection and co-infection (HIV) was confirmed.	Design of study was not reported. 176 drug addicts from 2 Prefectures were selected randomly.	No detailed age data reported (Born ≥ 1965). HCV Mono-infected 77% males. Additional data not reported.	Drug type, administration route. TID and risk behaviors were not reported.
[41]	August to October 2006. Drug users were recruited from the Liuzhou Methadone Clinic.	EC not reported. No details of selection of participants.	HCV antibody presence (ELISA). RNA reflex was conducted. HCV mono-infection and co-infection (HIV) was confirmed.	Sampling size of study not justified. Other analytical methods of sampling strategy not reported.	No age data was reported. Only whether individuals born on 1965 or after. HCV Mono-infected were 77% males.	Data was not available.
[42]	Information of FW dates only include year (2002–2003). Information on sites for recruitment and data collection was not reported.	Trained research staff determines characteristics on IDU were recruited from Liuzhou Methadone Clinic. The study served to determine dimension and characteristics of the condition.	Blood samples of HCV infection with ELISA. Samples were tested for HCV-RNA.	Sample size justification not reported. No data reported on design of the study or the sample framework.	Age average 32.4 (Range 17–61). 383 males (94%).	Type of drug and route was not available. Range years of drug use (1–18)
[43]	8–29 November 2002. Site of recruitment was study clinic in Xichang City.	EC: HIV seronegative. ≥ 18 y, injected within 3 months, willing to provide informed consent.	Samples were tested for antibodies with ELISA. RNA reflex was not conducted.	Community outreach and snowball sampling were methods for recruitment of IDU.	55% were <29 and 45% were ≥29 y o. 825.% were males	Type of drug and route were not reported. Frequency drug use, drug injection, shared injection. Sexual behavior (HIV or IDU partner, sex with men, casual, condom use, new partners and commercial sext.
[46]	August to December 2012. Place: Breves, Archipelago of Marajó, Pará, northern Brazil.	EC: older than 17 y. answer a questionnaire and blood sample for molecular and serologic markers.	Enzyme immunoassay (EIA) was used for testing HCV antibodies. RNA confirmation was conducted.	Cross sectional non probabilistic convenience sample was conducted. Snowball technique was used to sample IDU (areas of intense drug consumption).	Mean age 28.5 (sd 9.5) (range 18–51 years). 82.3% males	Cannabis (21.9%), cocaine paste (20.9%), cannabis and cocaine paste (13.4%), cocaine powder (18.2%), and oxi cocaine (25.6%). 14.4% had used ID at least once. TDU ≤3 years 32%.
[47]	2000–2001. Data from multicenter study AjUDE-Brazil II Project. Present study included data from 3 localities with intense drug use, trafficking and needle exchange program from University Bahia.	Residents in Salvador, Bahia. Informed consent. IDU: had injected drugs in the last 2 months. Ex-IDU had injected drugs in last 5 y, but not in last 2 months. Non-IDUs sniffed or smoked cocaine.	Anti-HCV immunoassays (abbot). RNA reflex performed.	Study size estimates were not reported. Sampling used snowball technique. Additional strategies for sampling enhance were not reported.	Mean age (sd) 26.6 ± 7.7. 93.8% (182/194) males	Participants were: 194 IDUs, 94 ex-IDUs and 95 non-IDUs. Data not available. Details on TID and risk behaviors were not reported.
[48]	August 2005- July 2006. Charitable, private and public drug treatment centers in Goiânia and Campo Grande, central-western Brazil.	EC: ≥ 18 y. IDU and/or Non-IDU; enrollment in a drug treatment center. Agreed to participate in the study.	Anti-HCV by ELISA was used for antibodies detection. Positive cases were confirmed with immunoblot test.	Cross sectional study conducted. Convenience sample drawn from treatment centers. All subjects informed and invited to study.	Data on age, sex and socioeconomic variables was not available for IDU group (separated from other groups).	IDU: report intravenous drug use; NIDU: reported never using ID and lifetime use of marijuana, cocaine (powder, crack, and “merla”), heroin, LSD, and ecstasy through other routes (sniffing, smoking, and ingestion).
[49]	1999–2001. Sites of recruitment were not clearly stated.	No specific EC were stated. Sources and methods for selection of participants. Methods of selection and recruitment of participants in Rio “drug scenes” were stated in other publication.	Immunoassay was used to detect VHC antibodies. RNA reflex was not conducted.	Cross sectional interview with structured questionnaire (WHO). Details on sampling frame were not provided only that “Drug scenes” were defined by a previous publication.	Mean age (sd) ST 27.4 (8.8) and LT 36.7 (8.3). 91.4% males.	Cocaine was the drug of choice. IDUS were classified in Long term (≥ 18 y) and short term users. Age first injection ST 20.6 (6.3), LT 18.5 (4). Duration of injection ST 2.2 (1.9), LT 16.1 (7.7) y.
[50]	Part of AJuDE-Brazil I project conducted (Blood samples) in 1998 (5 cities).	EC: a positive serology for HIV and/or HCV. Negative serology for both these infections. Exclusion: IDUs who presented positive serology HTLV-I/II.	Details of the HCV antibodies testing method were not reported/ RNA reflex was not conducted.	Study is part of a multi-center cross-sectional study. Sampling strategy was defined in main project. Details of sample frame were not reported.	Mean (sd) age 29 (8). 83% males. Skin color (49.5% white), read 89.5%, 69.7% have work.	No specific drug and route reported. Time of use > 10 years 28%. Sexual risks: debut age, sexual relation with other men. Life time active (passive) syringe sharing.
[51]	April 1991- December 1992. Recruitment in selected street settings and treatment clinics by IDU reference with mates.	Drug injection in the last 6 months, subjects >16 y, and provided informed consent to enter the study.	Anti-HCV by ELISA/RNA reflex not conducted.	Interviews conducted at usual meeting places. All IDUs who could be found and met inclusion criteria where recruited. Snowballing sampling was used.	WHO multicity questionnaire with 8 sections (including drug use pattern, sharing injection equipment and sexual behavior).	Data specific on type of drugs and route was not reported. Period of drug injection 9.4 (7.5) (range 1–37) y.
[52]	First project data collection (1994–1997) in 5 Brazilian cities. (n = 164)	Specific EC and selection of participants were not stated.	HCV antibodies were detected by a commercial immunoassay. Viral RNA test was conducted.	Details of study size were not reported. All interviewees received pre- and post-test counselling. HCV RNA-positive IDUs were referred to hospitals.	Age (years) first study 32.3 (7 .7) second study 32.2 (9 .8). 84.4% males	Data on specific type of drugs and route was not reported. Duration of IDU (years) first study 13.6 (8 .5) second study 9.6 (8 .7)
[53]	Dates of collection: 2000–2001. Sites were different ID scenes in 6 Brazilian cities.	EC and selection of IDUs were stated in previous studies AjUDE-Brazil I y II Projects.	ELISA diagnosis on filter paper. RNA reflex was not conducted.	A different part of the AjUDE-Brazil II project sample was reported for this study. IDUs were recruited by “outreach workers” by adapting the “targeted sampling” technique.	Sociodemographic data (sex, age, ethnicity, schooling, etc.) was collected. Age 29.2 y (7.9) 28.5 y (8.2) 83% males.	Cocaine injecting. TID: Mean age of first injecting drug use: 18.7 (5.1). Mean duration of injecting drug use: 9.8 (7.7). Sexual risks: condom use, men who report sex with other men, sex for drugs.
[54]	Dates of study: first semester of 1998. Two drug centers in Rio.	Specific EC were not stated. Coordinator contacted all the inpatients at each facility to explain the project's aims and procedures before mobile team collecting information on questionnaire, blood donation and clinical examination.	Anti-HCV was conducted with EIA/ RNA was conducted.	A detail of study size analysis was not reported. No individual compensation for time and effort was given to study participants. A check for data integrity was conducted.	Details on socioeconomic characteristics were not reported for subsample (n = 24) of IDUs was not reported.	Type of drug: cocaine injection 22/24 = 91.6%. Time of drug injecting: lifetime 24/24 = 100%.
[56]	Jun 2006-Oct 2007. Street drug users IDU and non-IDU/ Streets with IDU with help (information) from NGOs.	EC were not stated. Inclusion criteria: any drug use in the last 7 days and a willingness to be tested on-site for HCV and HIV. Methods of selection were not reported.	Rapid test kit, technique of test was not reported/RNA reflex was not conducted.	Study size and sampling methods for sampling strategy were not used because”…little is known about the characteristics of this group”	All sample current age 37.0 y (sd 8.21) Male: 522 (94.6%)	Type drug: Heroin 59.2%. Route: IDU was 95.3% of participants. All sample age when drug use began 24.3 years (SD 6.05). Risk behaviors: multiple sex partners: 59.4%, regular condom use 22.3%,
[55]	Jan 91 –May 92. Sites of recruitment were not reported.	EC details were not reported. Sources and methods for selection of participants were not reported.	Abbot HCV EIA 2^nd^ generation was used to detect HCV antibody. RNA reflex was not conducted.	Data on the type of study and methods for recruitment and follow up were not reported.	Data on demographic and additional information were not reported.	The unique information was on the type of patients including IDU: Other information was not reported.
[57]	Nov-Dec 2013. Places for recruitment of people who inject drugs (PWID) were no clearly reported. Initial seed seems to be given in different places of the city (Podgorica).	EC: >18 y, living in Podgorica at least 3 of 12 months before survey. Inject drugs for nonmedical purpose in a month preceding the survey. Additional methods for selection of participants were not described. Cross-sectional survey among using respondent driven sampling (RDS)	Anti-HCV was done with ELISA. RNA reflex was not conducted.	Recruitment initiated with 5 seeds (4 males 1 female) for different ages and places of residence. Seeds and participants were provided with 3 coupons to be used in recruiting other participants.	Median age was 32 (IQR 28–35) years. Men were 90.1%. Education, unemployment were reported.	Heroin and cocaine by injection. Administration route and use of drugs in past month. Risk behaviors: do not have a regular sexual partner, number of sexual partners in last year, condom use, frequency of injection, sharing syringes.
[58]	April- July 2008. Recruitment location for IDUs was not reported clearly.	EC: 18–51 y, injected drugs during the previous month, resided in Montenegro ≥ 3 months in the 12 months preceding the survey.	Anti-HCV test used ELISA/ RNA reflex was not conducted.	Participants received 2 monetary incentives one for face-to-face interview, blood sample, second for each person they successfully recruited into the study. Respondent driven sampling was used to recruit participants.	Mean age in years (sd) 28.6 (4.85). Male: 89.6%. Income, being arrested, treatment for addiction.	Age at first injection mean in years (sd) 22.6 8 4.6. Risk reported: shared syringes, frequency of injection.
[59]	September and October 2005. Interviews were conducted in fixed sites located in center of each city.	EC: Injecting drugs in previous 4 weeks, living in Belgrade or Podgorica, ≥18 years, willing to give a dried blood spot, did not participate in previous study.	Capillary blood onto absorbent paper by finger prick using disposable lancets for Anti-HCV cut off optical density. RNA reflex was not conducted.	2 cross sectional anonymous surveys. Respondent drive sampling (RDS) was used for recruitment. Pre-survey training and screening of potential participants were conducted.	27% were <25 y in Belgrade. 22% were females in Belgrade and 7% in Podgorica.	68% had ≤ 4 years of IDU. Risks reported: frequency of injection, reusing syringes, engaging in sex work.
[60]	February-April 2005. Sites: a facility from “Programa Compañeros” in Ciudad Juarez. Participants from Tijuana were enrolled during weekly trips to city neighborhoods.	EC: Injected illicit drugs in past month, confirmed injection stigmata, aged ≥18 y; ability to speak Spanish; willingness and ability to provide informed consent; and not previously interviewed for the study.	EIA was used and retesting with the same technique. RNA reflex was not conducted.	Cross sectional study of HIV and HCV infections factors. Respondent driven sampling was used to recruit participants. Additional details on sampling were reported.	Age at first injection Median 30 (IQR 16–25). 92% of males. Frequency of drinking alcohol.	Years of injecting Median ICR (8–19). Age at first injection. Risk behavior factors: sharing injecting devices.
[61]	September 2009- December 2010. Two survey sites one in each city, but a description of them was not made.	EC were not stated specifically. Consultations were conducted previous to Survey. RDS was used to recruit IDUs.	Anti-HCV Abbot/RNA was not conducted.	Multiplier method was used to estimate the size of IDU population. Two approaches were used to determine the multiplier. Sampling used seeds identified and selected to reflect diversity in residence, gender, levels of risk behavior, age and education.	Age distribution of participants % of males. Marital status and education were also collected.	Type drugs: Brown heroin, Subutext (opioid medical drug) and other drugs. Age at first injection drug use Risk behaviors: frequency of injection, shared needle at last injection, frequency sharing used syringes and injecting equipment, sexual risk factors.
[62]	Dates: 18 months from July 2003. Sites: Needle exchange program (NEP) from Initiative of health foundation. National center for addictions. Sofia Municipal center for addiction.	EC: Injected at least once in the last 6 months prior to interview, ≥14 years old. Sources and methods for selection of participants were not reported.	Details of the HCV antibodies testing method were not reported/ RNA reflex was not conducted.	Respondent driven recruitment (RDR) was used to enroll additional IDUs. Participants were given small monetary incentive to bring eligible individuals.	Mean age (sd) 25.9 y (5.7). Males: 79.4%. Ethnicity, current employment, being in jail, last month’s income.	Type and routes of administration were not reported. Time injecting drugs were not reported. Risks: sold sex for money or drugs, sexual partners in last 6 months, use condom with steady and non-steady partner, sex with IDU, sex with HCV infected.

Abbreviations: Drug users (DU), Eligibility criteria (EC), Hepatitis C virus (HCV), Injecting drug users (IDU), Interquartile range (IQR), methadone maintenance treatment (MMT), respondent drive sampling (RDS), Time injecting drugs (TID), written informed consent (WIC), Ribonucleic acid (RNA), enzyme-linked immunosorbent assay (ELISA), Antibody to hepatitis c virus (Anti-HCV).

The main outcome of the search was the prevalence of HCV infection. This was defined as the proportion of positive cases identified from the total number of individuals who were tested with any of the following Anti-HCV assays: ELISA, chemiluminescence, or any other test that detects antibody response to HCV antibody reactivity [26]. HCV cases were identified when the initial anti-HCV antibody immunoassay was positive. This test provides evidence of present or past infection. Additionally, a positive RNA-HCV test was conducted to indicate the presence of a current infection. If the results of both tests (anti-HCV and RNA-HCV) are available, the result of the first test is confirmed with a RNA-HCV test [27]. We defined “injection drug user” (IDU) as an individual who has been injecting any type of drug for more than six months prior to the study interview.

All studies that reported a prevalence of HCV infection and met the inclusion criteria were included in this analysis. Studies were included if they met the following criteria: 1) the study reported the prevalence of HCV in an IDU population, 2) the study was published after January 1990, and 3) the study was conducted in one of the upper middle income countries included in the search. The exclusion criteria were the following: 1) the purpose of the study was to identify individuals with HIV infection and not chronic HCV infection, and 2) the study only had male or only female subjects. Two members of the research team (VGG, LMS) independently read the published titles and abstracts, and selected the manuscripts that were reviewed in full. Differences in selected papers were resolved by consensus. One member of the team (VGG) read all the papers in full and excluded those with duplicated information based on the title and abstract, in agreement with the previously selected criteria.

We followed the STROBE statement guidelines for cross-sectional studies to retrieve and report information on studies included in the meta-analysis. The retrieval of information from observational studies tried to identify characteristics to the acronym PEOS (Population, Exposure, Outcome and Study type). Organization to report materials was made by information on study, methodology, and results. The study information we report includes dates and sites of data collection, while the methodology includes: eligibility criteria, type of tests for identifying HCV antibodies, use of RNA reflex testing for confirmation, sample size, and the analytical methods used to account for sampling strategy. In terms of results, we include whether studies report the demographic, clinical and social characteristics of participants, as well as information on type of drug use and mode of administration, time injecting drugs, and risks behavior for HCV infection.

The pooled HCV prevalence and its 95% confidence intervals (CIs) were estimated for each country and for all countries combined. The method used to estimate the pooling prevalence is to transform the prevalence to a variable that is not constrained to the 0–1 range and has an approximately normal distribution. The meta-analysis is conducted on the transformed proportions, using the inverse of the variance of the transformed proportion as the study weight, then the pooled transformed proportion and its CI are back transformed [28]. The Cochrane statistical Q-test was used with a level of <0.1 to evaluate the statistical heterogeneity of the results and the I^2 statistic with a range of values of 0 (no heterogeneity) to 100 (significant heterogeneity) [29]. We used the I^2 statistic to assess the level of heterogeneity within country and between countries. We also analyzed the pooled estimates of HCV prevalence according to the participants’ age and duration of IDU. The synthesis was performed with a random effects model (REM). The random effects model is considered more appropriate to estimate prevalence because it considers that the variability of the effect size is affected by the heterogeneity between studies and other aspects besides the variability within each study. The graphic representations of the results of the pooling results and their CIs were reported for each country in a Forest plots. The estimations were carried out using the Comprehensive meta-analysis V.3.3 software.

## Results

**Fig 1:**
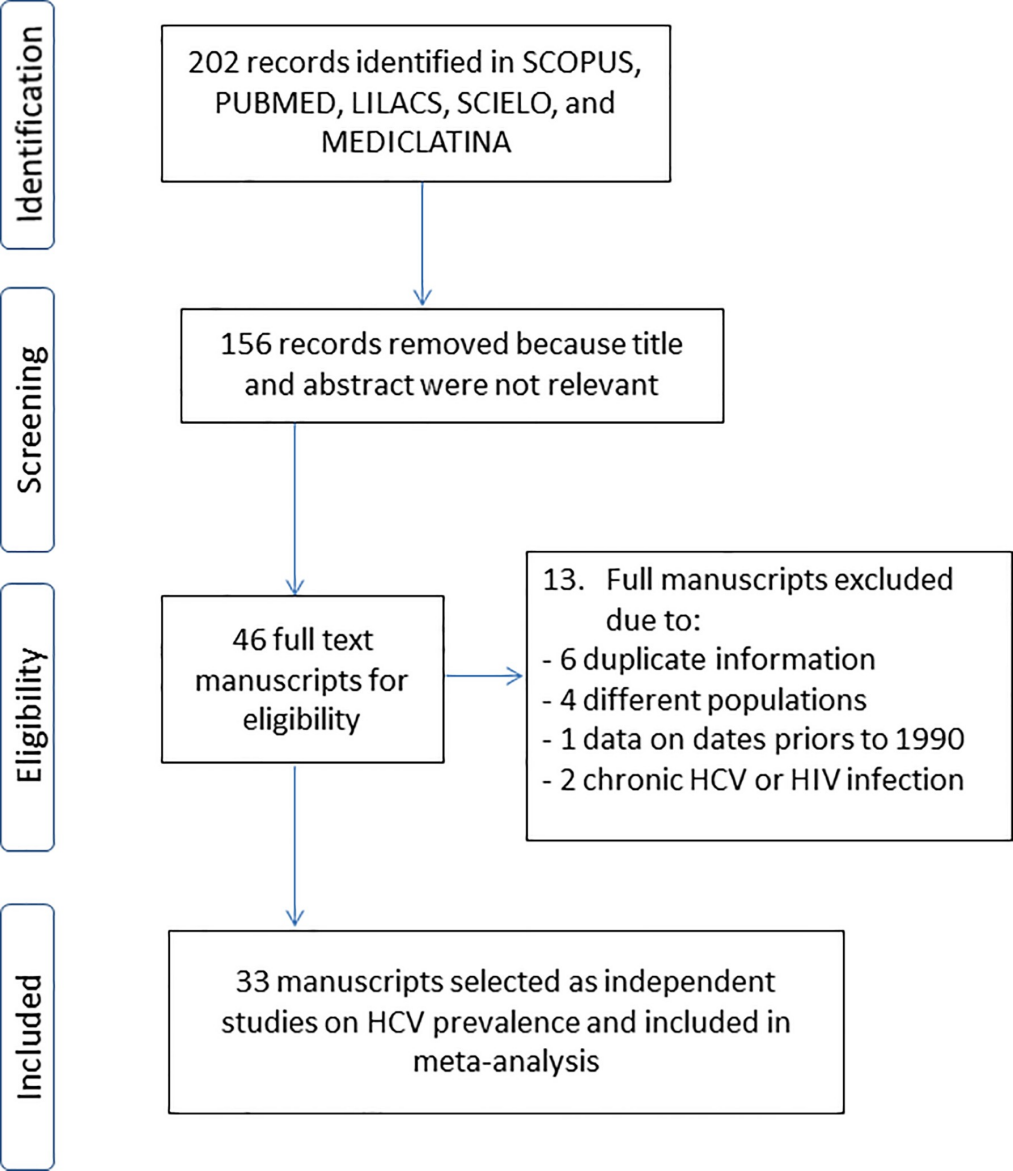
Search results and studies included in systematic review and meta-analysis.

Our search strategy identified 33 manuscripts from 202 potentially relevant records that were located based on the information provided in the title and abstract. A total of 156 documents were excluded because they did not meet the inclusion criteria (reviews, studies that were not conducted in upper middle income countries, did not include IDUs, or the article was not written in English or Spanish). The complete texts of 46 articles were reviewed to select those that would be included in the study. Thirteen articles were excluded due to the following: 1) duplicate information (n = 6), 2) a different study population (n = 4), 3) a focus on chronic HCV or HIV infection cases (n = 2), or 4) the study was conducted prior to 1990 (n = 1) (Fig 1).

Table 1 reports the study characteristics of the 33 publications we selected for this meta-analysis, including the prevalence of HCV infection among IDUs in seven upper middle income countries, from 1995 to 2015. Most studies were conducted in China (n = 16) [30–45], followed by Brazil (n = 9) [46–54], Malaysia (n = 2) [55, 56], and Montenegro (n = 3) [57–59], while only one study was identified for each of the following countries: Mexico, Mauritania, and Bulgaria (n = 3) [60–62]. The part of information corresponding to non-injection drug users (NIDUs) that was reported in two studies [48, 49], was excluded from the meta-analysis. But the agreeing with IDU was included. Additionally, we excluded the fragment of information regarding the prevalence of IDUs in the "Ajude-Brazil I" project [53] because it was previously reported in another article [50]. The part which was not repeated was included in the pooled analysis.

Most studies (n = 22) specified that they used a cross-sectional design, and even though nine studies did not explicitly state the design, it can be inferred as a cross-sectional survey. One study is described as a case-control design and another study conducted two surveys at different times. For some studies, the authors provide a brief description of the sampling strategy that was used. For example, six studies used respondent driven sampling, three used snow ball sampling, three used convenience sampling, and two applied a random selection of participants. One study used an existing database of serum samples and three studies did not provide details regarding the sampling strategy that was employed. In-person interviews with participants were conducted in 28 studies. Most studies (n = 19) provided information about the location where interviews were conducted and blood samples were collected. The most common recruitment sites were methadone clinics (n = 5), followed by detox centers, drug treatment centers, and study clinics (n = 9). Other recruitment areas, including program facilities or public streets, were described in five studies. Among the 29 studies that reported the proportion of males and females of the study sample 89% were males. Most of the studies included in our analysis report HCV prevalence based on results from the ELISA test (n = 20), followed by the chemiluminescence assays (Anti-HCV third generation) (n = 6). Seven studies do not report the type of assay used for HCV antibody reactivity testing. Only 8 studies report that confirmation with RNA-HCV test was conducted (Table 1).

The point prevalence estimates and CIs of the random effects model are 0.73 and 0.64–0.80, respectively, for all seven countries, and are highest for China (0.633; CI95%: 0.522–0.732), as compared to Brazil (0.396; CI95%:0.249–0.564), but they are not significantly different (p-value >0.05) (Table 2). Mexico (0.96) and Mauritania (0.973) also have a high prevalence, although these estimates are based on only one study per country. Malaysia (0.475) and Montenegro (0.416) have an intermediate prevalence (Table 2). The I^2 heterogeneity statistics indicate that heterogeneity was high in all countries, with a high associated p-value. The estimated Q statistic and corresponding p-value for the seven countries also shows that the heterogeneity among groups was high (Table 2).

**Table 2:** Meta-analysis HCV prevalence results for each country and by all countries.

Country	Number of studies	REM prevalence estimate (CI 95%)	I^2^ ^£^	p-value ^£^
China	16	0.633 (0.522–0.732)	99.14	0.000
Brazil	9	0.396 (0.249–0.564)	98.35	0.000
Malaysia	2	0.475 (0.177–0.792)	97.15	0.000
Montenegro	3	0.416 (0.237–0.621)	97.54	0.000
Mexico	1	0.96 (0.937–0.975)	0.00	1.00
Mauritius	1	0.973 (0.954–0.984)	0.00	1.00
Bulgaria	1	0.739 (0.707–0.768)	0.00	1.00
All countries ^¥^	33	0.729 (0.644–0.8)	98.97	0.000

^¥^ Heterogeneity among countries: Q = 585.36, p-value = 0.000

^£^ Heterogeneity among studies

**Fig 2:**
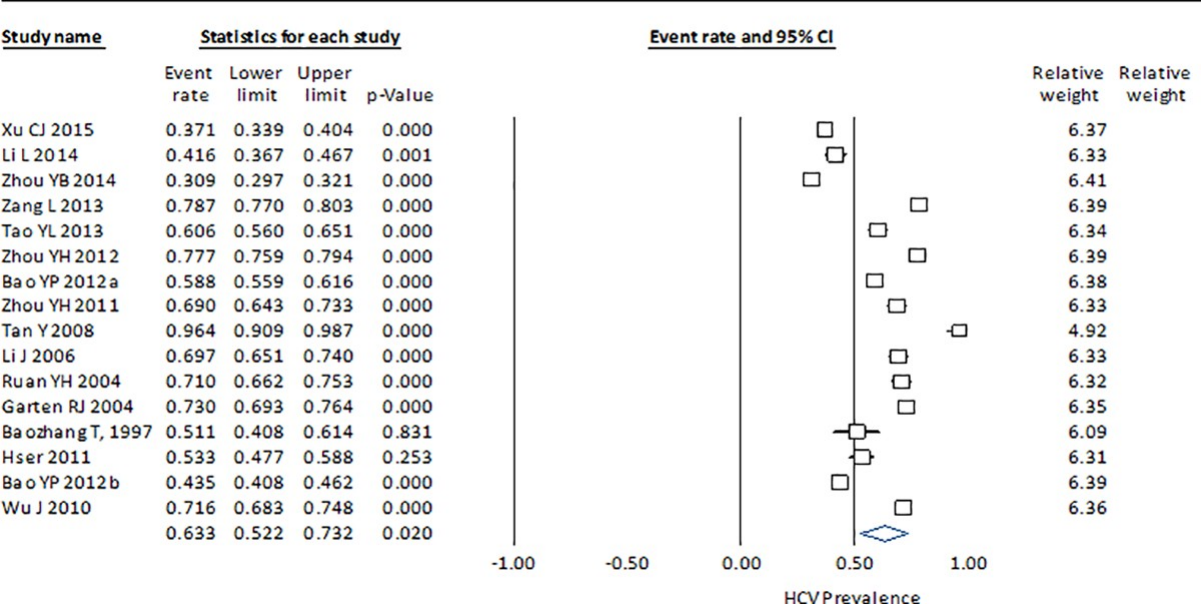
Forrest plot of random effects model to estimate HCV prevalence in IDU population in China.

**Fig 3:**
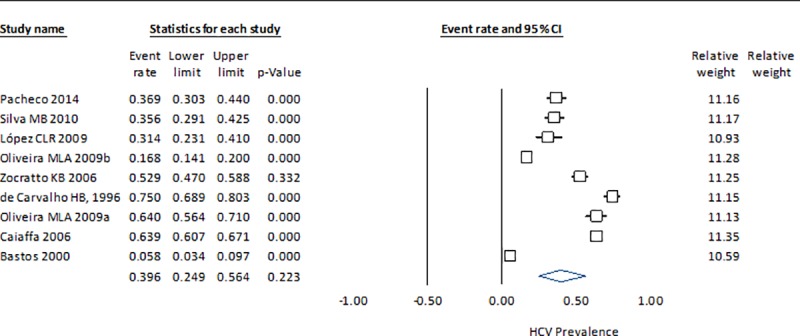
Forrest plot of random effects model to estimate HCV prevalence in IDU population in Brazil.

**Fig 4:**
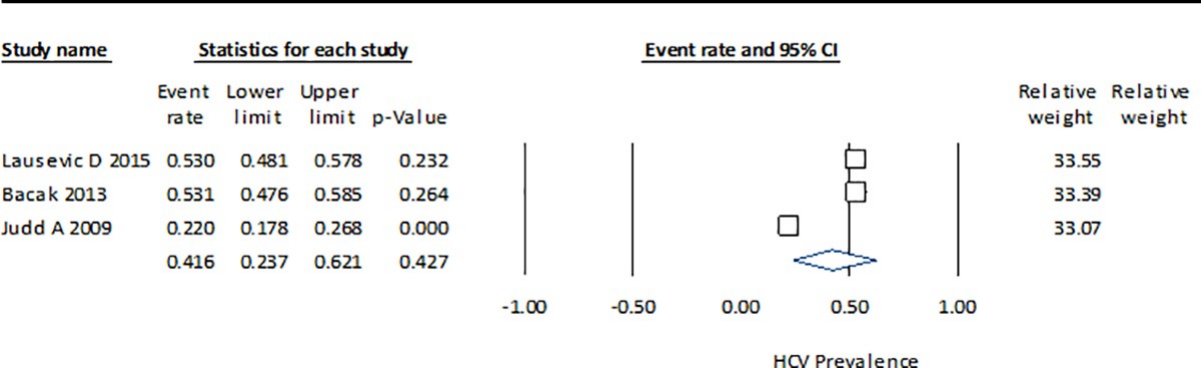
Forrest plot of random effects model to estimate HCV prevalence in IDU population in Montenegro.

**Fig 5:**
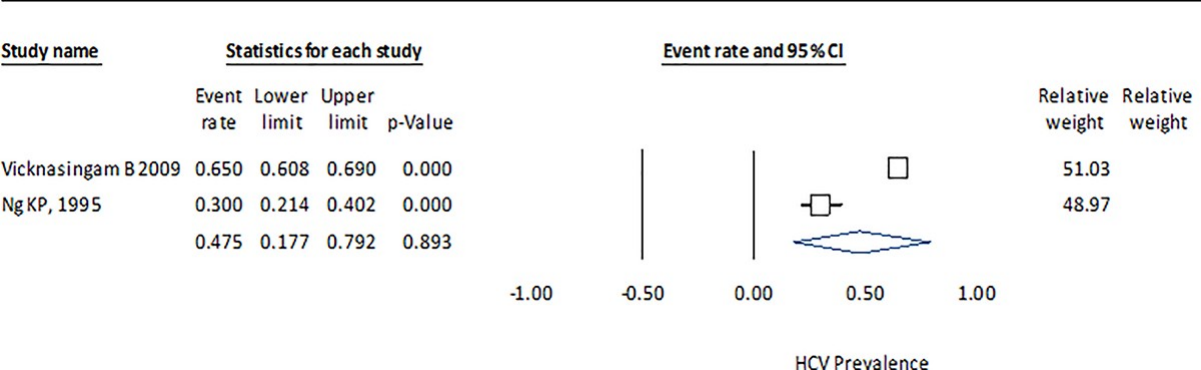
Forrest plot of random effects model to estimate HCV prevalence in IDU population in Malaysia.

The HCV prevalence results by country indicate that the measure of heterogeneity (Q statistic) for China is high, with a considerable range among studies (0.371–0.964) (Fig 2). In Brazil, the dispersion measure Q is lower, although in both cases the p-value is <0.001, which suggests that they do not share a common effect size (Fig 3). The estimate for Montenegro (0.416), which was calculated from three studies, is closer to the HCV prevalence in Brazil (0.396) than in China (0.633) and with a lower heterogeneity (Fig 4). The estimated prevalence of HCV in Malaysia (0.475) is similar to the one observed in Montenegro (Fig 5).

Although there is limited information regarding the age of participants in the 33 studies we included in this meta-analysis, Table 3 reports a possible association between age and prevalence of HCV. Of the six studies that indicate the participants’ age, over 50% are 40 years or older (SD 27%), with an average HCV prevalence of 70.5% (95%CI: 0.538–0.830). In another group of 17 studies that report mean age and standard deviation, the average age was 32 years (SD 8 years) and the corresponding prevalence is lower (0.536; 95%CI: 0.441–0.628). These findings suggest that the risk of becoming infected with HCV increases with age, which is to be expected.

**Table 3:** Results of meta-analysis. HCV prevalence by age and duration of IDU reported in 33 studies.

Groups of age of participants (years) ^¥^	Number of studies	Reported information	Prevalence (CI 95%)	I^2^ ^£^	Valor p ^£^
Average of % reported (sd)	6	> 40: 52% (sd 27%)	0.705 (0.538–0.830)	99.38	0.000
	1	> 29: 36% (only one Study)	0.569 (0.283–0.815)	98.42	0.000
	1	<25: 27% (only one study)	0.220 (0.178–0.268)	0.000	1.000
Average (sd)	17	32 (sd 8)	0.536 (0.441–0.628)	98.26	0.000
Median (min-max)	1	31(21–38)	0.716 (0.683–0.748)	0.000	1.000
Median (IQR)	1	32 (28–35)	0.53 (0.481–0.578)	0.000	1.000
Not reported	6	Age information of IDU not reported	0.682 (0.334–0.902)	98.75	0.000
All age groups	33	Different age parameters reported	0.520 (0.409–0.629)	99.57	0.000
Time of injection (years)^¶^	Number of studies	Reported information	Prevalence (CI 95%)	I^2^ ^£^	Valor p ^£^
Time range reported	1	≥ 10: 28%	0.529 (0.47–0.588)	0.00	1.0
	2	≥ 5: 40%	0.573 (0.28–0.823)	98.8	0.000
	1	> 2: 57%	0.730 (0.69–0.764)	0.00	1.00
	2	<4 (≤ 3): 50%	0.288 (0.165–0.453)	92.39	0.00
Time at first injection	1	26 years	0.371(0.339–0.404)	0.00	1.0
Average (sd)	9	11 (6)	0.613(0.488–0.724)	98.96	0.000
Median (IQR)	1	13 (8–19)	0.96(0.937–0.975)	0.00	1.00
Other parameter reported	7	Age at first injection (3 studies), range (1 study), all life injecting (1 study), confidence interval (1 study), time in months (1 study).	0.618(0.442–0.769)	99.10	0.000
No reported	9	Information of time of injecting not reported	0.550(0.388–0.703)	98.89	0.000
All groups	33	Different parameters reported	0.606(0.497–0.706)	99.57	0.000

^¥^ Heterogeneity among age groups: Q = 1,397, p-value = 0.000.

^¶^ Heterogeneity among time of injection groups: Q = 1,884, p-value = 0.000

^£^ Heterogeneity among studies

Table 3 also presents our findings regarding the relationship between time of IDU and HCV prevalence. Based on the information reported in nine studies, the average duration of IDU was 11 years (SD 6 years) and the prevalence of HCV was 0.613 (95%CI: 0.488–0.724). When 50% of the participants had a IDU duration of less than 3 or 4 years the HCV prevalence is significantly lower (0.28; 95%CI: 0.165–0.453).

## Discussion

Based on this systematic review of the literature, only two upper middle income countries (China and Brazil) have conducted nine or more studies that report the prevalence of HCV among IDU. Only two other countries have more than one study that reports the prevalence of HCV infection among IDU (Montenegro and Malaysia). The prevalence of HCV among IDU in China (0.633) is higher than in Brazil (0.396). The Montenegro and Malaysia studies have a similar prevalence (0.416) and (0.475), respectively, but a greater number of studies are needed to confirm the prevalence of HCV among IDUs in these countries. The overall HCV prevalence in the seven countries included in this review is high (0.729; 95%CI: 0.644–0.800), and we also found high heterogeneity in all our analyses. The values of the I2 statistics were high (> 95%) and the Q statistic among these countries was also significant. Our results suggest that most of the participants in the studies we reviewed were male, they began to inject drugs in their early 20s, and they continued to inject drugs for a long time. Our analysis also indicates that when IDUs are older and have injected drugs for a longer time, the prevalence of HCV is expected to be higher.

Variability within and between groups can be caused by different reasons such as the study location and methods of recruitment, sampling, diagnostic tests and time of data collection. However, we consider that the random effect model is an adequate analysis method to address the heterogeneity among studies.

The studies included in this review represent a more restricted group of countries, as compared to the reports that were included in other systematic reviews on the prevalence of HCV among IDU. In their review of the literature, Aceijas and Rhodes estimated the prevalence of HCV among IDU in different regions of the world, and found a greater variability [16]. The authors report the crude average of anti-HCV prevalence among IDUs: Latin America 0.08–0.9, Central Asia 0.1–1.0, South and Southeast Asia 0.34–0.9, East Asia and the Pacific 0.05–0.6. Brazil 0.39 to 0.69 [16]. In the particular case of Brazil our study found that REM estimates of prevalence are more similar than the crude prevalence range which is larger (0.05–0.75). Estimates with raw data in countries such as Mexico are based on a single study of patients with HIV and who in turn are IDUs [63]. In our review, we excluded studies that include IDU cases who are HIV positive, because we considered this may generate a bias in the prevalence estimate.

In another systematic review that included 77 countries from different areas of the world, Nelson et al. reported an overall prevalence of HCV among IDU in the range of 0.098–0.97 based on the midpoint reported by the studies [14]. The authors found that 26 of the 77 countries had a prevalence ranging from 0.6–0.8, while 12 countries had a prevalence greater than 0.8. The results of our study are not comparable to the review by Nelson et al., because we restricted our analysis to upper middle income countries, which narrows the range of HCV prevalence. Additionally, the estimates reported by Nelson et al. [14] are not the result of a synthesis model. Although we identified a very low HCV prevalence for a city in Brazil (0.058) and a very high prevalence in Bulgaria (0.973), the overall pooled prevalence estimated by the REM was 0.729 (CI95%: 0.644–0.800). Nonetheless each country or region may have different conditions that could result in a lower, intermediate or high prevalence.

Another systematic review that examined studies conducted in China, reports the prevalence of HCV among IDUs (the review considered only two studies written in English). Although this study included meta-analysis estimates, it does not report the prevalence estimates based on a random-effects model [21]. The authors report a measure of prevalence summary by region. The variations for the provinces were significant and the overall HCV prevalence was 0.614 (95% CI: 0.557–0.672). That estimate is similar to the one we predicted for this country (0.633; CI95%: 0.522–0.732). The authors reported a REM for HCV infection differences by sex, ethnic origin or being IDU vs non-IDU. In our study we could not conduct such type of analysis because of limited information regarding the participants' age and duration of IDU or other characteristics. Instead we conducted a REM for prevalence estimates and we found a great heterogeneity despite the concentration on selected countries by income level. This might be caused by factors such as: (1) age and sex of the participants, (2) availability of drugs, (3) quality of the data collection and information reported in each study, (4) IDU treatment practice, and (5) policies concerning IDU and treatment.

This study has some limitations, including that we did not have complete information about the age of participants and duration of IDU, which did not allow for an accurate analysis of these variables. Most studies only report the percentage of people older or younger than a specific age cutoff, and a limited number of studies report the average age and its standard deviation for the study population. Additionally, the duration of time as an IDU is reported for different periods and for different percentages of drug users. A second limitation is that the quality of the studies included in this review was not rated, as was done in a previous study [11], which allows for the evaluation of each study’s impact on the reported information. Another limitation is that we were unable to include studies that were published in languages other than English and Spanish, such as Chinese or Portuguese, which could have been relevant for this review.

Our systematic review and meta-analysis provides relevant information to characterize the prevalence of HCV infection among IDUs in upper middle income countries. Our study highlights that HCV infection is common among the IDU population, with a pooled prevalence of 0.729 (95% CI 0.644–0.800). The results of our systematic review and meta-analysis suggest that the prevalence of HCV in upper middle income countries can vary greatly, from a lower prevalence in Brazil to a higher prevalence in China. Despite the high heterogeneity in the meta-analysis, our results are relevant because they highlight the variability in the characteristics of the studies examined. This variability can be also a consequence of different methods and analysis techniques to estimate the prevalence of HCV among IDU in UMIC. Our study attempts to address some of the issues in measuring and estimating the prevalence of HCV among IDUs in UMIC. Although some studies in low- or middle-income countries may have limitations that prevent a more accurate characterization of HCV prevalence, we only included studies that specifically reported the prevalence of HCV in an IDU population. Having precise estimates of the prevalence of HCV infection in high risk groups is important so that national health systems can plan to meet the specific health needs of these vulnerable populations.

## Supporting information

S1 Table Search terms for HCV prevalence in UMIC. (DOCX)

S2 Table Countries, Cities, HCV prevalence and sample size reported in studies for meta-analysis. (DOCX)
