# Supplementary material for: Estimating the prevalence of hepatitis C among intravenous drug users in upper middle income countries: A systematic review and meta-analysis
Source: PLoS One. 2019 Feb 26;14(2):e0212558. doi: 10.1371/journal.pone.0212558 (PMC6391024; doi:10.1371/journal.pone.0212558)
Supplement: S1 Table — (DOCX) [file pone.0212558.s001.docx]

Table 1A Search terms for HCV prevalence in UMIC

| Search strategy (PubMed) |
| --- |
| Search (((((((((("Prevalence"[Mesh]) AND (("Hepatitis C"[Mesh]) OR "Hepatitis C Antibodies"[Mesh])) AND (("Substance-Related Disorders"[Mesh]) OR "Substance Abuse, Intravenous"[Mesh]))) AND ((((((((((((((((((((((((("azerbaijan") OR "belarus") OR "brazil") OR "botswana") OR "bulgaria") OR "china") OR "costa rica") OR "dominica") OR "gabon") OR "kazakhstan") OR "lebanon") OR "libya") OR "malaysia") OR "maldives") OR "mauritius") OR "mexico") OR "montenegro") OR "palau") OR "panama") OR "romania") OR "st lucia") OR "surinam") OR "turkey") OR "turkmenistan") OR "grenada"))) NOT "letter"[Filter])) NOT review[Filter])) NOT "chinese"[Filter] |
