# Supplementary material for: Estimating the prevalence of hepatitis C among intravenous drug users in upper middle income countries: A systematic review and meta-analysis
Source: PLoS One. 2019 Feb 26;14(2):e0212558. doi: 10.1371/journal.pone.0212558 (PMC6391024; doi:10.1371/journal.pone.0212558)
Supplement: S2 Table — (DOCX) [file pone.0212558.s002.docx]

#### Table 2A Countries, Cities, HCV prevalence and sample size reported in studies for meta-analysis.

| Author, Year | Country | Cities or Regions | Sample size | HCV prevalence |
| --- | --- | --- | --- | --- |
| Xu CJ, 2015 | China | Heben Province | 852 | 0.371 |
| Li L, 2014 | China | Ruili City | 370 | 0.415 |
| Zhou YB, 2014 | China | Yi prefecture | 5,628 | 0.309 |
| Zang L, 2013 | China | Guangdong | 2,296 | 0.787 |
| Tao YL, 2013 | China | Shanghai | 432 | 0.606 |
| Zhou YH, 2012 | China | Zhaotong, Qujing, Kaiyuan, Baoshan, Yingjiang | 2,080 | 0.777 |
| Bao YP, 2012a | China | Beijing | 1,121 | 0.588 |
| Bao YP, 2012b | China | Beijing | 1,327 | 0.435 |
| Zhou YH, 2011 | China | China Yunnan | 403 | 0.690 |
| Hser Y, 2011 | China | Shanghai, Kunming | 306 | 0.533 |
| Wu J, 2010 | China | Qing Yuan | 740 | 0.716 |
| Garten RJ, 2004 | China | Pingxiang, Binyang | 597 | 0.730 |
| Baozhang T, 1997 | China | Dehong and Lincang | 88 | 0.511 |
| Tan Y, 2008 | China | Guangxi | 112 | 0.96 |
| Li J, 2006 | China | Southwestern | 406 | 0.697 |
| Ruan YH, 2004 | China | Xichang | 379 | 0.71 |
| Pacheco SD, 2014 | Brazil | Breves | 187 | 0.369 |
| Silva MB, 2010 | Brazil | Salvador | 194 | 0.356 |
| López CLR, 2009 | Brazil | Goiania, Campo Grande | 102 | 0.314 |
| Oliveira MLA, 2009b | Brazil | Rio de Janeiro | 606 | 0.169 |
| Zocratto KB, 2006 | Brazil | Sao Paulo, Sorocaba, Sao José do Rio Preto , Itajaí and Porto Alegre | 272 | 0.529 |
| de Carvalho HB, 1996 | Brazil | Sao Paulo | 220 | 0.750 |
| Oliveira MLA, 2009a | Brazil | Rio de Janeiro | 164 | 0.642 |
| Caiaffa WT, 2006 | Brazil | Salvador, São José do Rio Preto, Florianópolis and Itajaí , and Porto Alegre and Gravataí | 857 | 0.640 |
| Bastos FI, 2000 | Brazil | Rio de Janeiro | 24 | 0.333 |
| Vicknasingam B 2009 | Malaysia | Penang, Kuala Lumpur, Johor Bharu, Kota Bahru, Kuantan | 526 | 0.654 |
| Ng KP, 1995 | Malaysia | Kuala Lumpur | 90 | 0.300 |
| Lausevic D 2015 | Montenegro | Podgorica | 402 | 0.531 |
| Bacak V, 2013 | Montenegro | Podgorica | 320 | 0.530 |
| Judd A, 2009 | Montenegro | Belgrado y Podgorica | 328 | 0.220 |
| White EF, 2007 | Mexico | Tijuana, Cd Juarez | 428 | 0.960 |
| Jonhston L, 2011 | Mauritius | Port louis, Curepipe | 511 | 0.973 |
| Vassilev ZP 2006 | Bulgaria | Sofia | 773 | 0.739 |
